# Childhood adversity and gestational diabetes: examining the mediating effect of BMI and depression through interventional mediation analysis

**DOI:** 10.1038/s41366-026-02080-9

**Published:** 2026-04-16

**Authors:** Megan Davies, Bianca De Stavola, Karoline Kragelund Nielsen, Peter Damm, Naja H. Rod

**Affiliations:** 1 Department of Public Health, University of Copenhagen, Copenhagen, Denmark; 2 Population, Policy & Practice Research and Teaching Department, UCL, London, UK; 3 Steno Diabetes Center Copenhagen, Copenhagen University Hospital, Copenhagen, Denmark; 4 Center for Pregnant Women with Diabetes, Department of Obstetrics, Rigshospitalet, Copenhagen, Denmark; 5 Department of Clinical Medicine, University of Copenhagen, Copenhagen, Denmark

**Keywords:** Gestational diabetes, Risk factors

## Abstract

**Background:**

Childhood adversity has been shown to increase the risk of gestational diabetes mellitus (GDM), though the mechanisms through which the risk is increased have yet to be examined.

**Methods:**

We used register data on all women born in Denmark and giving birth for the first time between 2004 and 2018, totalling 207,659 women. Interventional mediation analysis was used to estimate how two mechanistic pathways, depression and pre-pregnancy BMI, mediate the relationship between childhood adversity and GDM across five previously identified adversity trajectory groups.

**Results:**

4779 women were diagnosed with GDM between 2004 and 2018, corresponding to a 2.3% absolute risk of GDM. Compared to those who experienced low adversity in childhood, we estimated that those who experienced early material deprivation could have a 0.12% [95% CI 0.10, 0.14] reduction in absolute GDM risk by intervening on BMI and equalising its distribution to that of mothers in the low adversity group. We found no evidence of a reduction in risk if depression were similarly intervened on. For those in the high levels of adversity group no evidence of mediation was found.

**Conclusions:**

Intervening on pre-pregnancy BMI might potentially reduce the risk of GDM for mothers in some of the adversity groups, whereas intervening on depression seems to have little impact. The findings suggest that high adversity likely increases the risk of GDM through alternative mechanisms than BMI or depression.

## Introduction

GDM is characterised by glucose intolerance during pregnancy and is associated with an increased risk of perinatal complications and long-term health risks for both mother and offspring [1–3]. Exposure to stress has been found to impact the mother’s blood glucose levels during pregnancy and therefore increase the risk of GDM [4], though the mechanisms through which stress may increase GDM risk are not fully understood.

Stress experienced during childhood can have a profound impact on health across the life course. Childhood adversities, including poverty, loss within the family and abuse, have been associated with a higher risk of GDM [5–7]. Whilst the accumulation or severity of adversities tends to show a dose-response association with the risk for other health outcomes [8], the risk of GDM appears comparable across different types of adversity [7]. Chronic stress in early life may activate a response that releases stress hormones and inflammatory markers, increasing insulin resistance, as well as priming the stress response system for a stronger response to other stressors later in life, which may in turn increase GDM risk [9, 10].

Though the aforementioned mechanism provides a plausible theory for a direct association between childhood adversity and GDM, there are several other potential pathways that could explain such a link. Childhood adversity has repeatedly been shown to be associated with a higher lifetime risk of depression [11], which has been linked to the risk of developing GDM when diagnosed both before and during early pregnancy, again through an increase in stress responses affecting glucose regulation [12, 13]. Childhood adversity is also related to a higher body mass index (BMI) in adult life, which is a strong risk factor for GDM [14]. This may again be related to stress affecting biological systems that increase an individuals susceptibility to body fat [15], as well as behavioural responses to stress, such as using food as a coping mechanism. The effect of pre-conception depression on GDM has been found to persist even after adjusting for BMI, suggesting that depression may increase the risk of GDM through mechanisms independent of BMI status [5]. Furthermore, the bi-directional relationship between obesity and depression observed in previous studies [16] means it is not always possible to ascertain a sequential relationship between these factors, and therefore, we seek to understand the separate pathways through which pre-pregnancy depression and BMI may mediate the relationship between adversity and GDM.

Understanding the potential mechanisms of the effect of childhood adversity on GDM is essential to identify additional points for intervention. Using nationwide register-based life-course data in Denmark, we aim to quantify the effect of exposure to childhood adversities on GDM risk, which operates through the mediating pathways of depression and BMI. Using interventional mediation analysis, we aim to disentangle the interventional direct effect of childhood adversity on GDM, the interventional indirect effects of depression and BMI, and the combined total effect.

## Methods

We used data from the DANish LIFE course (DANLIFE) cohort, which includes all children born in Denmark from 1980 onwards [17]. All Danish citizens are assigned a unique personal identification number at birth, allowing different registers to be linked, which cover health and administrative information, as well as the ability to link parental and sibling data. For this study, we identified women in the DANLIFE cohort born between 1980 and 2001, who were resident in Denmark until their 16th birthday and registered in the Danish Medical Birth Register [18] as giving birth for the first time during the period 2004 until 2018. We did not include women with pre-existing diabetes, and included only women aged 16 and above to ensure the adversities during the entire childhood period could be measured, giving a final study population of 207,659 women between 2004 and 2018.

### Childhood adversities

DANLIFE includes twelve different adversities captured across three dimensions; family dynamics, material deprivation, and loss or threat of loss [17]. Previous research using the DANLIFE cohort identified five trajectory groups of adversity exposure, which account for adversity type, accumulation and duration of exposure [19] (see Supplementary Table S1 for specific adversities included): ‘Low adversity’ is characterised by low levels of adversity across all dimensions throughout childhood, ‘Early life material deprivation’ and ‘Persistent material deprivation’ capture material deprivation in early childhood and through childhood and adolescence, respectively, ‘Loss or threat of loss’ captures high annual rates of parental or sibling severe somatic illness or death across childhood and adolescence, and finally ‘High adversity’ is characterised by high and increasing annual rates of adversity across all dimensions throughout childhood and adolescence.

### GDM

GDM diagnosis was identified from the Danish National Patient Register, using the International Classification of Diseases 10th Revision (ICD-10) code O24.4. Clinically, women with GDM are diagnosed by a risk-factor-based selective screening approach throughout Denmark followed by a 75 g oral glucose tolerance test [20].

### Depression and BMI

Diagnosed depression was retrieved through the National Patient Register, which records depression diagnosed through both outpatient and inpatient hospital contact. Depression was included as a binary variable, and we included both primary and secondary diagnoses (ICD8: 296, 296.2, 298, 300.4, ICD10: F32-33) received prior to GDM diagnosis. Pre-pregnancy BMI was retrieved through the Medical Birth Register, where height and weight are recorded at the first antenatal visit. BMI was treated as a continuous variable and only available in the register from 2004, hence we include cases from then. In a sensitivity analysis, we include antidepressant use alongside diagnosed depression to capture more cases of depression treated outside of hospital contact, retrieved from the Danish National Patient Registry (ATC: N06A).

### Covariates

Potential confounders included parental country of origin (European origin/non-European origin), parental type 2 diabetes (yes/no), and the age category of the participant’s mothers when the participants themselves were born (<20 years/20–30 years/>30 years). Parental origin was classified as non-European descent if one or both parents had a nationality from a country outside of Europe, North America, Australia, and New Zealand. Parental history of type 2 diabetes was retrieved through the Danish Adult Diabetes Registry (2005–2018) (ICD8: 250/ICD10: E11) and the Danish National Patient Register (1977–2018) [21], and supplemented with information on purchased prescriptions of oral anti-diabetic drugs and insulin from the Danish National Prescription Registry, based on the protocol by Carstensen et al. [22]. We additionally adjusted for the participant's age at the time of delivery, captured from the Danish Medical Birth Register and included as a continuous variable. Family adversity is also highly linked with parental education and being born small for gestational age and/or preterm, as familial adversity could affect the health of the child already in utero. These factors may be mediators on the effect of childhood adversity on GDM rather than confounders, therefore, we decided to only adjust for the effects of being born small for gestational age (intrauterine growth below the 10th percentile of age- and sex-specific reference curves) [23], preterm birth and the participant’s parents' educational level at their own time of birth (low <10 years/medium 10–12 years/high >12 years) [24] in supplementary analyses.

### Statistical analysis

We first tested the association between childhood adversity and depression and BMI by running logistic and linear regression analyses, respectively. To examine the mediating effect of childhood adversity on GDM through BMI and depression, we used interventional mediation analysis [25]. Unlike natural effects that conceptualise the comparison of “cross-world” interventions [26], interventional effects consider the impact of shifting the distribution of the mediator(s) to that seen in the unexposed, allowing us to estimate how much of the risk of an outcome could be reduced if we intervened on the mediator. In this case, we estimate for each adversity group how much of the absolute risk of GDM could be reduced if the distribution of BMI (or depression) were shifted to that of unexposed individuals (i.e. those in the low adversity group). When interventional effects are applied to multiple mediators as in this case, the total effect is decomposed into: 1) the interventional direct effect, 2) the interventional indirect effect through depression and BMI, respectively, and 3) an additional term, which is the remainder after the direct and indirect effects are added together. This term is generally zero when the mediators are conditionally independent (given exposure and confounders); when they are not, it captures the effect of the exposure on the outcome that acts via interactions with and among the mediators (and which is often referred to as “the dependence of the two mediators” and is usually very small) [25]. When an order between the two mediators cannot be presumed, the interventional direct effect captures the result of only intervening on the adversity group without changing the distributions of the mediators, while the indirect effect via each mediator captures the result of intervening on the distribution of that mediator in turn, but keeping the other mediator, if downstream from the first, fixed. This implies that the interventional indirect effects represent the change in risk when the distribution of BMI (and any variable that lies on the causal path from adversity to BMI, i.e. its predecessors) is shifted to that of the individuals who experienced low adversity, and similarly for the interventional indirect effect of depression. The interventional indirect effects therefore calculate the risk that would remain for each adversity group if the distribution of the relevant mediator were shifted from that of the exposed to the unexposed group. We ensured that we met the model-based assumptions for interventional effect analysis, including measurement of confounding, correct model specification using g computation, and consistency for both exposure and mediator [25].

Interventional mediation analysis was performed in R via the package *Intmed* and expressed on the risk difference scale. Due to high proportions of missing data on BMI, multiple imputation was performed internally using the Intmed package, which uses Multiple Imputation by Chained Equations (MICE). Only BMI was imputed, with the imputation model including all variables and outcome and mediators used in the mediation analysis as predictors. Overall, there were 3.14% of records with missing data, and therefore 5 datasets were imputed via the *MICE* package in R. We present the results of the MICE-based analysis and include the complete records analysis in Supplementary material.

## Results

Amongst the study population, 4779 women were diagnosed with GDM during pregnancy, corresponding to a 2.3% risk in the population. Descriptive characteristics for individuals by adversity group are shown in Table 1.

**Table 1:** Background characteristics of the study population by adversity trajectory group in %.

	Low adversity (48.0%)	Early life material deprivation (22.6%)	Persistent Deprivation (17.9%)	Loss or threat of loss (8.2%)	High adversity (3.4%)
GDM	99,881	47,009	37,206	17,043	7147
No	98.0%	97.5%	97.3%	97.4%	97.5%
Yes	2.0%	2.5%	2.7%	2.6%	2.5%
Missing	0%	0%	0%	0%	0%
SGA					
No	82.7%	81.7%	78.5%	79.6%	72.6%
Yes	13.2%	15.1%	17.1%	17.2%	23.6%
Missing	4.1%	3.2%	4.4%	3.2%	3.8%
Participants’ mothers’ age at birth					
*<*20 years	1.4%	4.7%	8.3%	4.8%	12.8%
20-30 years	74.2%	79.3%	75.3%	70.9%	71.3%
*>*30 years	24.4%	16.0%	16.4%	24.2%	15.9%
Missing	*<*1%	*<*1%	*<*1%	*<*1%	*<*1%
Parental type 2 diabetes					
No history	91.3%	89.8%	85.7%	86.1%	87.7%
History	8.7%	10.2%	14.3%	13.9%	12.3%
Missing	0%	0%	0%	0%	0%
Parental education					
Low	11.4%	25.7%	37.4%	26.9%	57.1%
Medium	51.1%	56.2%	48.2%	50.2%	34.2%
High	37.2%	17.8%	13.7%	22.5%	7.6%
Missing	*<*1%	*<*1%	*<*1%	*<*1%	1.1%
Parental origin					
Non-European	0.4%	1.2%	4.5%	1.6%	1.0%
European	99.5%	98.8%	95.5%	98.3%	99.0%
Missing	*<*1%	0%	0%	*<*1%	0%
BMI					
Mean (SD)	23.98 (4.80)	24.50 (5.28)	24.48 (5.43)	24.33 (5.25)	24.14 (5.68)
Missing	2.85%	2.99%	3.68%	3.23%	4.22%
Maternal age					
Mean (SD)	27.1 (3.6)	25.9 (3.8)	25.9 (4.0)	25.4 (4.1)	24.1 (4.1)
Missing	0%	0%	0%	0%	0%
Depression					
	1.1%	1.3%	1.5%	1.7%	2.4%
No	98.9%	98.7%	98.5%	98.3%	97.6%
Depression and antidepressant use					
No	85.0%	82.2%	81.5%	78.4%	73.1%
Yes	15.0%	17.8%	18.5%	21.6%	26.9%

**Fig. 1:**
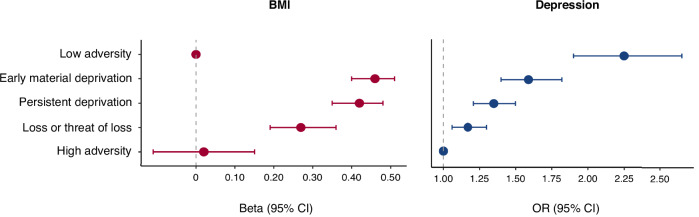
Adjusted beta coefficients (kg/m^2^) and odds ratios and 95% CI for association between childhood adversity and BMI (left) and depression (right). Models were adjusted for maternal age, parental type 2 diabetes history, parental origin, and age of participant's mother at birth.

We initially estimated prospective associations between childhood adversity on depression and BMI separately, shown in Fig. 1. The results show a higher risk of being diagnosed with depression in all adversity trajectory groups compared to low adversity, with the highest associated risk being observed amongst those with high adversity (OR 2.25 [95% CI 1.90–2.66]). Childhood adversity was also associated with a higher BMI, most strongly with the material dimensions of adversity groups and particularly for early material deprivation (difference: 1.57 kg/m^2^ [1.49–1.67]) compared to low adversity.

### Interventional mediation analysis

The results of the interventional mediation analysis shown in Table 2 are expressed as estimated risk differences (%). Overall, the results show that being assigned to each adversity group versus the reference low adversity group increases the GDM risk by about 0.5% (estimated total effects: 0.43% (95% CI 0.27–0.60), 0.39% (0.22–0.57), 0.53% (0.28–0.78) and 0.52% (0.17–0.93) for early life material deprivation, persistent deprivation, loss or threat of loss, and high adversity, respectively). These effects could be reduced by about 0.10% for the first three groups by intervening on the distribution of BMI (0.12% (0.10–0.14), 0.11% (0.09–0.13), and 0.08% (0.06–0.11)) but not for the high adversity group (0.00 (−0.04–0.03)). This corresponds to the proportion mediated through BMI as 28% for early life material deprivation, 28% for persistent deprivation, 16% for loss or threat of loss, and only 2% for high adversity.

**Table 2:** Estimated interventional effects (shown as risk differences (%)) of childhood adversity on GDM through depression and BMI and 95% CI.

Effect	Risk difference (%)
Early life material deprivation vs low adversity	
Indirect effect through depression	0.00 (0.00, 0.00)
Indirect effect through BMI	0.12 (0.10, 0.14)
Direct effect	0.31 (0.15, 0.47)
Remainder	0.00 (−0.02, 0.02)
Total effect	0.43 (0.27, 0.60)
Persistent deprivation vs low adversity	
Indirect effect through depression	0.00 (0.00, 0.01)
Indirect effect through BMI	0.11 (0.09, 0.13)
Direct effect	0.28 (0.11, 0.45)
Remainder	0.00 (−0.02, 0.02)
Total effect	0.39 (0.22, 0.57)
Loss or threat of loss vs low adversity	
Indirect effect through depression	0.01 (0.00, 0.01)
Indirect effect through BMI	0.08 (0.06, 0.11)
Direct effect	0.44 (0.20, 0.69)
Remainder	0.00 (−0.03, 0.03)
Total effect	0.53 (0.28, 0.78)
High adversity vs low adversity	
Indirect effect through depression	0.01 (0.00, 0.02)
Indirect effect through BMI	0.00 (−0.04, 0.03)
Direct effect	0.52 (0.17, 0.93)
Remainder	0.00 (−0.05, 0.05)
Total effect	0.52 (0.17, 0.93)

In contrast, intervening on the distribution of depression does not appear to have any impact: for each of the adversity groups, there would be very little reduction in the risk of GDM if depression were shifted to that of those in the low adversity group. These two sets of results imply that either depression is downstream from BMI but does not have a detectable separate impact on GDM, or it is upstream of BMI, but its impact is nearly completely captured by BMI.

For the high adversity group, the total difference in risk could not be explained by depression or BMI, and therefore most of the risk difference of GDM for high adversity compared to low adversity comes from other mediating pathways. We found no remaining residual effect from the dependence of BMI and depression for any of the adversity groups.

The complete records analysis overall corroborated the results based on the imputed samples, though with slightly larger direct and total interventional effects for early life material deprivation and loss or threat of loss, and slightly lower for high adversity (Supplementary Table 2). Sensitivity analyses including antidepressant use showed similar results across the adversity groups (Supplementary Table 3), though the inclusion of SGA and parental education attenuated the results (Supplementary Table 4).

## Discussion

In a large nationwide cohort, we aimed to identify and quantify two potential mechanisms through which childhood adversity may increase the risk of GDM. It has previously been shown that exposure to any type of adversity during childhood is associated with a higher risk of GDM [7]. We add to this evidence by showing that the risk of GDM could potentially be reduced if BMI were ‘intervened’ on, i.e. shifted to that of an individual from the low adversity group, for all groups except for the high adversity group. We found that there would be very little effect on the risk of GDM across the adversity groups from intervening on depression.

In line with previous results of the effect of childhood adversity on BMI, we found there to be little association between high adversity and BMI in this sample [27], and shifting BMI to that of the low adversity group would have little impact on reducing the risk of GDM in the high adversity group. BMI contributed to a greater proportion of the total effect in the other three trajectory groups, particularly for those exposed to early material deprivation and persistent deprivation. Poverty has previously been found to be associated with a higher risk of overweight and obesity, often due to families having less access to healthier food choices, limited opportunities for physical activities, and living in areas with a higher proportion of unhealthy food venues [28]. Chronic levels of stress in childhood, compounded with limited resources, may instead for some individuals exposed to high adversity, be linked to a lower as opposed to higher BMI.

Despite studies showing that depression increases the risk of GDM, we estimated that for each adversity group, the interventional effect on depression is not likely to reduce the risk of GDM. Pre- and early pregnancy depression has been found to be associated with an increased risk of GDM, however there is limited evidence that treating depression would decrease GDM risk [29]. In a sensitivity analysis where we included antidepressant use to capture more cases of depression, the results were unchanged. Though our results may suggest intervening on depression would not have an effect, it may be that by including diagnosed depression, these individuals are likely to have received some degree of treatment, through pharmacological interventions or talking therapies, with depression therefore having been ‘intervened’ on already. These findings may also be indicative of a distinct mechanism through which adversity increases the risk of GDM. The long-term effect of stress in childhood may have a long-lasting impact on biological functioning, which may then directly increase an individual's risk of GDM [30], independent of depression, and could explain the increased risk directly from adversity, particularly amongst those who experienced high adversity.

This study is, to the best of our knowledge, the first to examine potential mediating factors between childhood adversity and GDM risk, and importantly demonstrates the mechanisms of GDM risk for some groups. Our application of interventional mediation analysis allowed us to estimate the potential reduction in the risk of GDM associated with each adversity group if these two hypothesised pathways were shifted to that of an individual who experienced low adversity. Interventional effects are particularly useful if the sequential relationship of the mediators is unknown, as it still allows the indirect effects through each pathway to be estimated, albeit with some care needed in interpretation, enabling us to show how the mechanism between childhood adversity and GDM risk differs between the adversity groups. We were also able to show that for some potentially more vulnerable individuals exposed to adversity, intervening on BMI and depression is likely to have a small impact on reducing the risk of GDM. Structural measures focusing on housing and income support, as well as providing support for bereaved families, may be promising in preventing childhood adversity, which may then in turn improve health outcomes including reducing GDM prevalence along with its associated health risks.

Though our use of register data allowed us to capture several objectively measured adversities, there are some limitations that should be noted. We were unable to include direct measures of abuse or sexual violence, though our inclusion of foster care is likely to capture the most severe cases of abuse within the home. We were unable to establish the sequential relationship between BMI and depression, which may have given more insight into the direction of their relationship and their effect on GDM. Due to a lack of data from GP visits in the registers, we were only able to include cases of depression diagnosed through hospital contacts, which is therefore likely to capture more severe cases of depression in this study population. Though we include antidepressant use in sensitivity analyses, we cannot be certain these were used in cases of depression, as antidepressants can be prescribed for other conditions. Finally, the risk-factor-based screening protocol for GDM used in Denmark [20] may mean that some women did not attend screening calls and therefore the cases of GDM in the registries may be underestimated.

In conclusion, we found evidence that intervening on BMI may reduce the risk of GDM to some extent for all but the high adversity group. Intervening on depression would most likely not be associated with a reduced risk of GDM for any of the adversity groups. Our findings indicate that, particularly for those groups who experienced differing rates of deprivation during childhood, a higher BMI may explain some of the effect of adversity on GDM risk. For those who experienced high levels of adversity, the increased risk of GDM may come directly from prolonged exposure to stress in childhood, or from mechanisms that have yet to be identified. Preventing childhood adversity itself through upstream social interventions may be more effective in decreasing GDM and its associated health risks.

## Supplementary information

Supplementary material

## Data Availability

The data contains personally identifiable and sensitive information, and cannot be made publicly available in accordance with the Act on Processing of Personal Data. Inquiries regarding access to the DANLIFE cohort granted by Statistics Denmark and the Danish Health Data Authorities should be directed to the PI of DANLIFE Professor Naja Hulvej Rod (nahuro@sund.ku.dk).

## References

[1] Clausen TD, Mathiesen ER, Hansen T, Pedersen O, Jensen DM, Lauenborg J, et al. High prevalence of type 2 diabetes and pre-diabetes in adult offspring of women with gestational diabetes mellitus or type 1 diabetes: the role of intrauterine hyperglycemia. Diab Care. 2008;31:340–6. 10.2337/dc07-1596 18000174

[2] Ovesen PG, Jensen DM, Damm P, Rasmussen S, Kesmodel US. Maternal and neonatal outcomes in pregnancies complicated by gestational diabetes. a nation-wide study. J Matern Fetal Neonatal Med. 2015;28:1720–4. 25228278 10.3109/14767058.2014.966677

[3] Kramer CK, Campbell S, Retnakaran R, Retnakaran RaviRetnakaran R. Gestational diabetes and the risk of cardiovascular disease in women: a systematic review and meta-analysis. Diabetologia. 2019;62:905–14. 30843102 10.1007/s00125-019-4840-2

[4] Horsch A, Kang JS, Vial Y, Ehlert U, Borghini A, Marques-Vidal P, et al. Stress exposure and psychological stress responses are related to glucose concentrations during pregnancy. Br J Health Psychol. 2016;21:712–29. 27169679 10.1111/bjhp.12197

[5] Schoenaker DAJM, Callaway LK, Mishra GD. The role of childhood adversity in the development of gestational diabetes. Am J Prev Med. 2019;57:302–10. 31353162 10.1016/j.amepre.2019.04.028

[6] Mason SM, Tobias DK, Clark CJ, Zhang C, Hu FB, Rich-Edwards JW. Abuse in childhood or adolescence and gestational diabetes: a retrospective cohort study. Am J Prev Med. 2016;50:436–44. 26547539 10.1016/j.amepre.2015.08.033 PMC4801767

[7] Davies M, van Houten CS, Bengtsson J, Elsenburg LK, Kragelund Nielsen K, Andersen GS et al. Childhood adversity and the risk of gestational diabetes: a population-based cohort study of nulliparous pregnant women. Diabetic Med. 2023. 10.1111/dme.15242. 10.1111/dme.15242 37845190

[8] Bengtsson J, Elsenburg LK, Andersen GS, Larsen ML, Rieckmann A, Rod NH. Childhood adversity and cardiovascular disease in early adulthood: a Danish cohort study. Eur Heart J. 2023;44:586–593. 10.1093/eurheartj/ehac607 36375818

[9] Pervanidou P, Chrousos GP. Metabolic consequences of stress during childhood and adolescence. Metabolism. 2012;61:611–9. 22146091 10.1016/j.metabol.2011.10.005

[10] Berens AE, Jensen SKG, Nelson CA. Biological embedding of childhood adversity: From physiological mechanisms to clinical implications. BMC Med. 2017;15. 10.1186/s12916-017-0895-4. 10.1186/s12916-017-0895-4 PMC5518144 28724431

[11] Pusch D, Dobson KS. Childhood adversity and adult depression: the protective role of psychological resilience. Child Abus Negl. 2017;64:89–100. 10.1016/j.chiabu.2016.12.012 28056359

[12] Byrn M, Penckofer S. The relationship between gestational diabetes and antenatal depression. J Obstet Gynecologic Neonatal Nurs. 2015;44:246–55. 10.1111/1552-6909.12554 25712378

[13] Hinkle SN, Buck Louis GM, Rawal S, Zhu Y, Albert PS, Zhang C. A longitudinal study of depression and gestational diabetes in pregnancy and the postpartum period. Diabetologia. 2016;59:2594–602. 27640810 10.1007/s00125-016-4086-1 PMC5101167

[14] Hedderson MM, Gunderson EP, Ferrara A. Gestational weight gain and risk of gestational diabetes mellitus. Obstet Gynecol. 2010;115:597. 20177292 10.1097/AOG.0b013e3181cfce4f PMC3180899

[15] Kühnel A, Hagenberg J, Knauer-Arloth J, Ködel M, Czisch M, Sämann P, et al. Stress-induced brain responses are associated with BMI in women. Commun Biol. 2023;6:1031. 10.1038/s42003-023-05396-8 PMC10567923 37821711

[16] Vittengl JR. Mediation of the bidirectional relations between obesity and depression among women. Psychiatry Res. 2018;264:254–9. 29655968 10.1016/j.psychres.2018.03.023

[17] Bengtsson J, Dich N, Rieckmann A, Rod NH. Cohort profile: the DANish LIFE course (DANLIFE) cohort, a prospective register-based cohort of all children born in Denmark since 1980. BMJ Open. 2019;9. 10.1136/bmjopen-2018-027217. 10.1136/bmjopen-2018-027217 PMC6756429 31542736

[18] Bliddal M, Broe A, Pottegård A, Olsen J, Langhoff-Roos J. The Danish medical birth register. Eur J Epidemiol. 2018;33:27–36. 29349587 10.1007/s10654-018-0356-1

[19] Rod NH, Bengtsson J, Budtz-Jørgensen E, Clipet-Jensen C, Taylor-Robinson D, Andersen A-MN, et al. Trajectories of childhood adversity and mortality in early adulthood: a population-based cohort study. Lancet. 2020;396:489. 32798491 10.1016/S0140-6736(20)30621-8

[20] Jensen DM, Mølsted-Pedersen L, Beck-Nielsen H, Westergaard JG, Ovesen P, Damm P. Screening for gestational diabetes mellitus by a model based on risk indicators: a prospective study. Am J Obstet Gynecol. 2003;189:1383–8. 14634573 10.1067/s0002-9378(03)00601-x

[21] Jørgensen ME, Kristensen JK, Husted GR, Cerqueira C, Rossing P. The Danish adult diabetes registry. Clin Epidemiol. 2016;8:429. 27843339 10.2147/CLEP.S99518 PMC5098513

[22] Carstensen B, Rønn PF, Jørgensen ME. Prevalence, incidence and mortality of type 1 and type 2 diabetes in Denmark 1996-2016. BMJ Open Diabetes Res Care. 2020;8. 10.1136/bmjdrc-2019-001071. 10.1136/bmjdrc-2019-001071 PMC7265004 32475839

[23] Maršál K, Persson PH, Larsen T, Lilja H, Selbing A, Sultan B. Intrauterine growth curves based on ultrasonically estimated foetal weights. Acta Paediatr. 1996;85:843–8. 8819552 10.1111/j.1651-2227.1996.tb14164.x

[24] Jensen VM, Rasmussen AW. Danish education registers. Scand J Public Health. 2011;39:91–94. 21775362 10.1177/1403494810394715

[25] Vansteelandt S, Daniel RM. Interventional effects for mediation analysis with multiple mediators. Epidemiology. 2017;28:258–65. 27922534 10.1097/EDE.0000000000000596 PMC5289540

[26] Rijnhart JJM, Lamp SJ, Valente MJ, MacKinnon DP, Twisk JWR, Heymans MW. Mediation analysis methods used in observational research: a scoping review and recommendations. BMC Med Res Methodol. 2021;21. 10.1186/s12874-021-01426-3. 10.1186/s12874-021-01426-3 PMC8543973 34689754

[27] Elsenburg LK, Rieckmann A, Bengtsson J, Lange T, Baker JL, Sørensen TIA et al. Early childhood adversity and body mass index in childhood and adolescence: linking registry data on adversities with school health records of 53,401 children from Copenhagen. Int J Obes. 2023. 10.1038/s41366-023-01355-9. 10.1038/s41366-023-01355-9 PMC10599995 37626127

[28] Ziol-Guest KM, Duncan GJ, Kalil A. Early childhood poverty and adult body mass index. Am J Public Health. 2009;99:527–32. 19106427 10.2105/AJPH.2007.130575 PMC2661458

[29] Arafa A, Dong JY. Depression and risk of gestational diabetes: a meta-analysis of cohort studies. Diabetes Res Clin Pract. 2019;156. 10.1016/j.diabres.2019.107826. 10.1016/j.diabres.2019.107826 31449873

[30] Danese A, Caspi A, Williams B, Ambler A, Sugden K, Mika J, et al. Biological embedding of stress through inflammation processes in childhood. Mol Psychiatry. 2011;16:244–6. 20157309 10.1038/mp.2010.5 PMC4212809

