# Supplementary material for: Childhood adversity and gestational diabetes: examining the mediating effect of BMI and depression through interventional mediation analysis
Source: Int J Obes (Lond). 2026 Apr 16;50(8):1706–11. doi: 10.1038/s41366-026-02080-9 (PMC13461335; doi:10.1038/s41366-026-02080-9)
Supplement: Supplementary file 1 [file 41366_2026_2080_MOESM1_ESM.docx]

**Supplementary material**

**Supplementary Table s1.** Dimensions that cover specific childhood adversities, their definitions and registers retrieved from. These cover the adversities across the five trajectory groups of adversity identified by Rod et al. (1). Experts in stress, child health and child psychology were included as part of a panel that decided on the dimensions of childhood adversity.

| **Dimension** | **Adversity** | **Definition** | **Registers** |
| --- | --- | --- | --- |
| **Material deprivation** | **Family poverty** | Family income <50% of the median national family income in a given year | The Income Statistics Register |
|  | **Parental long-term unemployment** | Either or both parents being unemployed for at least 12 months | The Integrated Database for Labour Market Research |
| **Loss or threat of loss** | **Death of a parent** | Death of a parent | The Danish Civil Registration System |
|  | **Death of a sibling** | Death of a sibling | The Danish Civil Registration System |
|  | **Parental somatic illness** | A parent diagnosed with a disease included in the Charlson comorbidity index | The Danish National Patient Register |
|  | **Sibling somatic illness** | A sibling being diagnosed with one of the seven somatic illnesses most commonly linked to mortality in children aged 0–18 years in Denmark:  malignant neoplasm; congenital anomalies of the heart and circulatory system; congenital anomalies of the nervous system; cerebral palsy; epilepsy; cardiomyopathy; congenital disorders of lipid metabolism | The Danish National Patient Register |
| **Family dynamics** | **Foster care** | Being placed in out-of-home care | The Register of Support for Children and Adolescents |
|  | **Parental psychiatric illness** | A parent admitted as an inpatient for at least 1 day to a psychiatric hospital or ward, with a primary diagnosis related to psychiatric illness (excluding primary diagnoses related to alcohol and drug abuse) | The Danish Psychiatric Central Research Register; The Danish National Patient Register |
|  | **Sibling psychiatric illness** | A sibling admitted as an inpatient for at least 1 day to a psychiatric hospital or ward with a primary diagnosis related to psychiatric illness | The Danish Psychiatric Central Research Register; The Danish National Patient Register |
|  | **Parental alcohol abuse** | A parent diagnosed with a disease that is related to alcohol abuse, or purchasing a drug that has been prescribed and used in the treatment of alcohol dependence | The Danish Psychiatric Central Research Register; The Danish National Patient Register; The Danish National Prescription Registry |
|  | **Parental drug abuse** | A parent diagnosed with a disease that is related to drug abuse, or purchasing a drug that has been prescribed and used in the treatment of drug addiction | The Danish Psychiatric Central Research Register; The Danish National Patient Register; The Danish National Prescription Registry |
|  | **Maternal separation** | The mother no longer registered as sharing an address with a partner | The Danish Civil Registration System |

References to Supplementary Table 1

1. Rod NH, Bengtsson J, Budtz-Jørgensen E, Clipet-Jensen C, Taylor-Robinson D, Andersen A-MN, et al. Trajectories of childhood adversity and mortality in early adulthood: a population-based cohort study. The Lancet. 2020 Aug 15;396(10249):489–97.

**Supplementary table s2.** Complete records of main analysis

| **Early life material deprivation v low adversity** | **Complete records** |
| --- | --- |
| Indirect effect through depression | 0.00 (0.00, 0.00) |
| Indirect effect through BMI | 0.12 (0.10, 0.14) |
| Direct effect | 0.32 (0.16, 0.49) |
| Remainder | 0.00 (-0.02, 0.02) |
| Total effect | 0.44 (0.28, 0.62) |
| **Persistent deprivation v low adversity** |  |
| Indirect effect through depression | 0.00 (0.00, 0.01) |
| Indirect effect through BMI | 0.11 (0.09, 0.12) |
| Direct effect | 0.26 (0.08, 0.43) |
| Remainder | 0.00 (-0.02, 0.02) |
| Total effect | 0.37 (0.18, 0.55) |
| **Loss or threat of loss v low adversity** |  |
| Indirect effect through depression | 0.01 (0.00, 0.01) |
| Indirect effect through BMI | 0.07 (0.05, 0.10) |
| Direct effect | 0.45 (0.21, 0.68) |
| Remainder | 0.00 (-0.03, 0.03) |
| Total effect | 0.53 (0.28, 0.78) |
| **High adversity v low adversity** |  |
| Indirect effect through depression | 0.01 (0.00, 0.02) |
| Indirect effect through BMI | 0.01 (-0.03, 0.04) |
| Direct effect | 0.53 (0.17, 0.90) |
| Remainder | 0.00 (-0.05, 0.05) |
| Total effect | 0.54 (0.17, 0.93) |

**Supplementary table s3.** Sensitivity analysis of antidepressant use and diagnosed depression as mediator

| **Early life material deprivation v low adversity** | MICE |
| --- | --- |
| Indirect effect through depression | 0.02 (0.02, 0.03) |
| Indirect effect through BMI | 0.12 (0.10, 0.14) |
| Direct effect | 0.29 (0.13, 0.44) |
| Remainder | 0.00 (-0.02, 0.02) |
| Total effect | 0.43 (0.27, 0.60) |
| **Persistent deprivation v low adversity** |  |
| Indirect effect through depression | 0.03 (0.02, 0.04) |
| Indirect effect through BMI | 0.11 (0.09, 0.13) |
| Direct effect | 0.24 (0.08, 0.41) |
| Remainder | 0.00 (-0.02, 0.02) |
| Total effect | 0.39 (0.21, 0.56) |
| **Loss or threat of loss v low adversity** |  |
| Indirect effect through depression | 0.06 (0.04, 0.07) |
| Indirect effect through BMI | 0.07 (0.05, 0.10) |
| Direct effect | 0.39 (0.16, 0.64) |
| Remainder | 0.00 (-0.03, 0.04) |
| Total effect | 0.53 (0.28, 0.78) |
| **High adversity v low adversity** |  |
| Indirect effect through depression | 0.10 (0.08, 0.14) |
| Indirect effect through BMI | 0.00 (-0.03, 0.04) |
| Direct effect | 0.41 (0.06, 0.79) |
| Remainder | 0.01 (-0.04, 0.05) |
| Total effect | 0.53 (0.16, 0.92) |

**Supplementary table s4.** Sensitivity analysis including small for gestational age and parental education

| **Early life material deprivation v low adversity** | MICE |
| --- | --- |
| Indirect effect through depression | 0.00 (0.00, 0.00) |
| Indirect effect through BMI | 0.06 (0.05, 0.08) |
| Direct effect | 0.20 (0.05, 0.37) |
| Remainder | 0.00 (-0.02, 0.02) |
| Total effect | 0.27 (0.11, 0.44) |
| **Persistent deprivation v low adversity** |  |
| Indirect effect through depression | 0.00 (0.00, 0.00) |
| Indirect effect through BMI | 0.02 (0.01, 0.04) |
| Direct effect | 0.10 (-0.06, 0.27) |
| Remainder | 0.00 (-0.02, 0.02) |
| Total effect | 0.13 (-0.04, 0.30) |
| **Loss or threat of loss v low adversity** |  |
| Indirect effect through depression | 0.01 (0.00, 0.01) |
| Indirect effect through BMI | 0.03 (0.01, 0.05) |
| Direct effect | 0.33 (0.09, 0.58) |
| Remainder | 0.00 (-0.03, 0.03) |
| Total effect | 0.36 (0.12, 0.62) |
| **High adversity v low adversity** |  |
| Indirect effect through depression | 0.01 (0.00, 0.02) |
| Indirect effect through BMI | -0.09 (-0.13, -0.06) |
| Direct effect | 0.22 (-0.11, 0.59) |
| Remainder | 0.00 (-0.04, 0.05) |
| Total effect | 0.13 (-0.19, 0.50) |
